# Spike Timing and Reliability in Cortical Pyramidal Neurons: Effects of EPSC Kinetics, Input Synchronization and Background Noise on Spike Timing

**DOI:** 10.1371/journal.pone.0000319

**Published:** 2007-03-28

**Authors:** Victor M. Rodriguez-Molina, Ad Aertsen, Detlef H. Heck

**Affiliations:** 1 Department of Neurobiology and Biophysics, Institute of Biology III, Albert-Ludwigs-University, Freiburg, Germany; 2 Facultad de Medicina, Universidad Autónoma del Estado de Morelos, Cuernavaca, Morelos, México; 3 Bernstein Center for Computational Neuroscience, Albert-Ludwigs-University, Freiburg, Germany; 4 Department of Anatomy and Neurobiology, University of Tennessee Health Science Center, Memphis, Tennessee, United States of America; National Insitutes of Health, United States of America

## Abstract

In vivo studies have shown that neurons in the neocortex can generate action potentials at high temporal precision. The mechanisms controlling timing and reliability of action potential generation in neocortical neurons, however, are still poorly understood. Here we investigated the temporal precision and reliability of spike firing in cortical layer V pyramidal cells at near-threshold membrane potentials. Timing and reliability of spike responses were a function of EPSC kinetics, temporal jitter of population excitatory inputs, and of background synaptic noise. We used somatic current injection to mimic population synaptic input events and measured spike probability and spike time precision (STP), the latter defined as the time window (Δt) holding 80% of response spikes. EPSC rise and decay times were varied over the known physiological spectrum. At spike threshold level, EPSC decay time had a stronger influence on STP than rise time. Generally, STP was highest (≤2.45 ms) in response to synchronous compounds of EPSCs with fast rise and decay kinetics. Compounds with slow EPSC kinetics (decay time constants>6 ms) triggered spikes at lower temporal precision (≥6.58 ms). We found an overall linear relationship between STP and spike delay. The difference in STP between fast and slow compound EPSCs could be reduced by incrementing the amplitude of slow compound EPSCs. The introduction of a temporal jitter to compound EPSCs had a comparatively small effect on STP, with a tenfold increase in jitter resulting in only a five fold decrease in STP. In the presence of simulated synaptic background activity, precisely timed spikes could still be induced by fast EPSCs, but not by slow EPSCs.

## Introduction

### Background

Nerve cells exchange information through brief electrical signals called spikes or action potentials, which are triggered by fluctuations in the neuron's membrane potential. Each spike is communicated via synapses to thousands of other neurons, causing small changes in the receiving neuron's membrane potential called post-synaptic potentials (PSPs). Neocortical neurons receive several thousand PSPs per second, which causes their membrane potential to constantly fluctuate. The amplitudes and time courses of these fluctuations depend on the types of synapses, their number, spatial distribution and synchronization, and on the level of “background” synaptic activity. Understanding the “translation” of synaptically driven fluctuations of membrane potential to spike events is of key importance to our understanding of computational processes in the brain.

### Methodology/Principal findings

Here we studied how membrane potential fluctuations trigger spikes in cortical layer V pyramidal cells. We injected fluctuating currents into the cells to simulate natural membrane potential fluctuations driven by various input events. Our findings show that the shape of postsynaptic potentials not only determines the temporal precision and reliability with which cortical pyramidal cells generate spikes, but also how spike generation is affected by the amplitude and temporal jitter of population inputs and by the overall level of synaptic input activity.

### Conclusions/Significance

Our results show that the temporal precision with which cortical layer V pyramidal neurons fire spikes and how spike temporal precision is influenced by noise, temporal jitter and input amplitude depends critically on the shape of EPSPs. Surprisingly, the time constant of the EPSP decay phase turned out to have the strongest influence on spike temporal precision and reliability of spike firing. This has interesting functional implications since the decay time constant depends on the dendritic location of the synapse and on the active and passive membrane properties of the postsynaptic neuron. Our findings, thus, suggest that precisely timed spike patterns, at least in the close-to-threshold regime, may be preferentially communicated via proximal synaptic terminals and between neurons with small membrane time constants.

## Results

There are two main aspects to a neuron's spike response to a stimulus: 1) the reliability or probability with which a spike response is generated, i.e. the fraction of stimuli triggering a spike when the stimulus is repeated several times, and 2) the temporal precision with which the spike follows the stimulus, i.e. the width of the time window within which spike responses occur. The importance of the timing aspect of spike generation has become obvious since accumulating experimental evidence suggests that spike time precision may be an important parameter in the processing of information in cortical networks [1]–[3]. The precise timing of spikes depends partly on the time course of the membrane potential fluctuations [4]. Such fluctuations reflect, in part, the ongoing activity from the network impinging on the neuron [5] and the resulting activation of the underlying excitatory postsynaptic currents (EPSCs) and their kinetics. Several mechanisms, such as passive and active dendritic properties [6]–[13], spatial as well as temporal summation [14]–[17], and synaptic plasticity [18] affect the time course of the membrane potential fluctuations that ultimately reach the soma. Moreover, changes in ongoing network activity affect neuronal membrane resistance and time constant through synaptic conductances, and hence, also control the shape of excitatory postsynaptic potentials (EPSPs) [19]–[22].

The relationship between EPSPs or EPSCs shape and the timing of evoked spikes have been described in previous works [23]–[27]. These authors report two main findings: First, the smaller the half width (HW) of EPSPs, the higher the temporal precision of the spike response. Second, the larger the amplitude of an excitatory membrane potential trajectory, the higher the temporal precision of the evoked spike response.

Here, we investigated in detail how spike time precision depends on the time course of the somatic membrane potential changes induced by simulated EPSCs. We independently varied the time constants of the EPSC's rise and decay to study the role each of these play in controlling the precision and reliability of response spikes. Our baseline measurements were all taken at threshold level. We then explored in detail how changes in EPSC amplitude, number of EPSCs in an input volley, degree of synchronization of inputs, and the presence of noise affects STP. We injected currents that simulated the physiological time courses of EPSCs [27]–[31]. All experiments were performed in neocortical layer V pyramidal neurons *in vitro* in whole cell patch configuration.

Several studies suggested that the high variability of spike firing *in vivo* is due to the variability in the activation of postsynaptic terminals during synaptic transmission [4], [20], [32]–[34]. Other studies have pointed to “intrinsic noise” sources (ion channels stochasticity) as a cause for spike timing variability [34]–[41]. We investigated how fluctuating currents mimicking stochastic background synaptic inputs influence STP. Fluctuating currents were added to the input signal either during or just prior to EPSC stimulation (then ending with EPSP onset). Comparing the two protocols allowed us to discriminate between effects of membrane potential noise riding on top of the EPSP and the effects of having ion channels in different states of activation at the time of input arrival. The influence of the two different sources of background noise is discussed in detail. Overall, our findings suggest that spike time precision at threshold level is most sensitive to the EPSC decay time.

### EPSC decay time influences spike time precision

**Figure 1:**
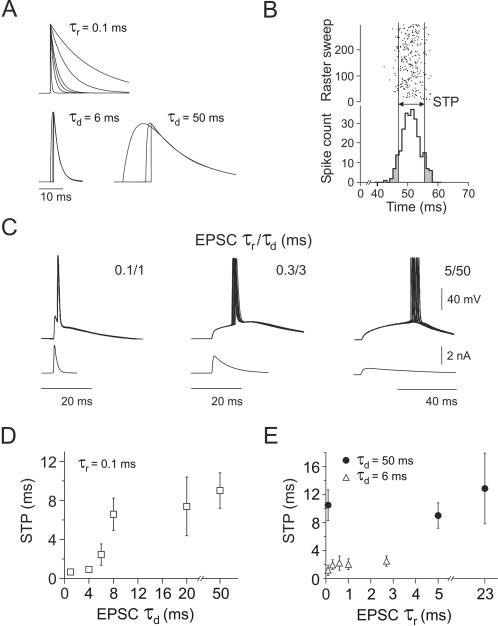
Generation of different EPSC shapes by independently varying rise and decay times and measurement of spike time precision. *A*, Time varying currents, produced by a beta-function mimicking EPSCs with constant rise time (0.1 ms) and variable decay times (upper left) or constant decay times (lower left: 6 ms, lower right: 50 ms) and variable rise times. *B*, Raster and peri-stimulus time histogram (PSTH, bin width = 1 ms) depicting spike responses to 300 presentations of an identical compound EPSC stimulus aligned on EPSC onset. Raster points represent the occurrence of action potentials. STP defined as the central 80% area of the PSTH (*white region*). *C*, Example raw data traces (12 traces superimposed) of spike responses elicited to the stimulation with different compound EPSCs: Left to right τ_rise_/τ_decay_ 0.1/1, 0.3/3, 5/50 ms. *D,E*, Mean STP as a function of EPSC decay and rise time (mean±s.e.m; *n* = 10 cells). *D*, STP as a function of EPSC decay time constant showed two levels of precision: high STP for τ_decay_ values≤6 ms and low STP for τdecay≥6 ms. *E*, EPSC rise time had no significant effect on STP in combinations with the two different decay times (6 and 50 ms, *open* and *solid symbols*) shown.

To evaluate the role of EPSC rise and decay times in spike time precision (STP) at spike threshold level, we repeatedly injected synchronized volleys of 100 simulated EPSCs into the soma of layer V neocortical pyramidal neurons *in vitro* to elicit spike responses (Figure1 *A,C*). We compared the spike responses elicited by compound EPSCs with constant rise time (0.1 ms) and variable decay times or, alternatively, variable rise times and constant decay time (6 or 50 ms) (Figure 1*A,D,E*). The injection of the compound EPSCs was made from an average resting potential of −65±0.37 mV (mean±s.e.m.), the mean voltage depolarization averaged over all suprathreshold compound EPSCs kinetics was 25.2±0.79 mV (mean±s.e.m.) (Figure 1*C*). Similar compound amplitudes have been observed in intracellular recordings *in vivo* [4].

The spike time precision, defined as the width of the centered time window that held 80% of all response spikes (Figure1 *B*) [42], was measured off line (cf. Methods). Compounds of EPSCs with decay time constant smaller than 6 ms (which in the following are termed “fast EPSCs”) induced responses with a high STP (≤2.45 ms; *n* = 10), while larger EPSCs decay times (termed “slow EPSCs”) produced temporally less precise responses (Δt≥6.58 ms; *n* = 10). There was a sharp transition in STP between EPSCs with decay times of 4 and 8 ms, suggesting that STP was a sigmoidal function of EPSC decay time, with a steep rise between 4 and 8 ms (Figure 1*D*). This sharp transition in STP separates EPSCs into two classes: those with decay times shorter than ∼6 ms elicit spikes at high temporal precision, whereas those with decay times longer than ∼6 ms elicit spikes only at low temporal precision. Why the STP – decay time function is this steep and why at this particular point remains do be determined. The fact that these measurements were made at threshold level certainly plays a role, as we will later see that EPSCs with long decay times do yield precise spike timing when their amplitude is increased to above threshold level. Surprisingly, changing the EPSC rise time within a physiological range while keeping the decay time constant had only very little effect on STP compared to the effects of changes in decay time while keeping the rise time constant. Even when the rise time exceeded the average membrane time constant (mean = 12.9 ms, s.e.m. = 0.08 ms), no significant changes in STP were observed (Figure 1*E*). These results suggest that the two levels of spike time precision documented in figure 1*E* are a consequence of the associated EPSC decay phase, see Figure 1*D*.

**Figure 2:**
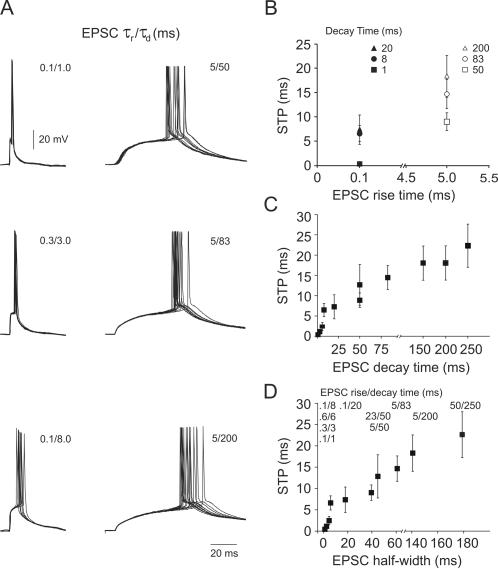
Physiological EPSCs induce spike responses with a temporal precision that is mostly determined by EPSC decay time. *A*, Action potentials induced by the repeated somatic injection of physiological compound EPSCs. Each plot shows 12 traces of raw data superimposed. The *left column* shows spike responses to compound EPSCs with fast kinetics (τ_rise_/τ_decay_ 0.1/1, 0.3/3 and 0.1/8 ms). The *right column* shows responses to compound EPSCs with slow kinetics (τ_rise_/τ_decay_ 5/50, 5/80 and 5/200 ms). *B*, Mean STP is plotted as a function of EPSC rise time. Note that the same rise time could be combined with up to three different decay times to produce different EPSC shapes. *C*, STP plotted as a function of EPSC decay time constant. Same data as shown in *B*. *D*, STP plotted as a function of EPSC width at half amplitude. Same data as shown in *B*.

We measured spike responses to a variety of physiological EPSC shapes with values of rise and decay times reported in the literature (Figure *2A,B*). Compounds of EPSCs with τ_rise_/τ_decay_ of 0.1/1, 0.3/3 and 0.6/6 ms induced rapid transitions of the somatic membrane potential, and were associated with temporally precise spike responses with Δt<2.45 ms (mean = 1.35 ms, s.e.m. = 0.30 ms). By contrast, EPSCs with τ_rise_/τ_decay_ of 0.1/8, 0.1/20, 5/50, 23/50, 5/83, 5/200 and 50/250 ms resulted in correspondingly slower changes in membrane potential and elicited temporally less precise spike responses with a average STP of Δt = 7.65 ms, s.e.m. = 0.37 ms (Figure 2*A*). We found only a weak correlation between STP and rise time. On the other hand, the strong correlation between STP and both decay time and half width suggests that details of the decay phase may be less relevant and that the main effect can be explained by the time span the membrane potential spends close to spike threshold. This time span is directly proportional to the EPSC width at half amplitude, which is almost entirely determined by the decay time constant as demonstrated by the fact that STP vs. decay time and STP vs. half width are almost identical functions (Figure 2*C, D*). As described in detail below, EPSC decay time was also strongly correlated with the spike delay after EPSC onset: EPSCs with short decay times triggered spikes at a short delay and vice versa.

### Influence of compound EPSC amplitude on spike time precision

**Figure 3:**
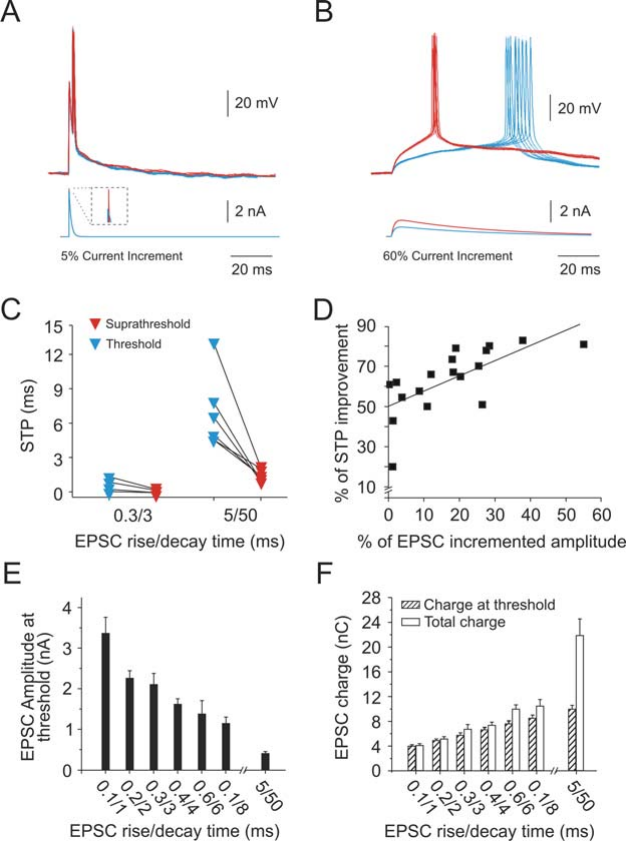
Increasing compound EPSC amplitude increases STP. For both fast and slow compound EPSCs increasing the amplitude improved STP. However, slow compounds required an ∼40% increase in amplitude to improve STP by 50%, whereas only ∼5% amplitude increase was required to achieve the same improvement with fast compound EPSCs. *A,B*, Action potential responses induced by compound EPSCs at threshold (*blue*) and suprathreshold (*red*) level. For each case in *A,B*, 10 raw data traces are shown superimposed. *A*, Action potentials induced by a compound EPSC of τ_rise_/τ_decay_ 0.3/3 ms. Red traces are responses to stimuli with a 5% current increment above threshold level; inset shows a zoom of the tip of the trace. *B*, Spike responses induced by a compound EPSC of τ_rise_/τ_decay_ 5/50 ms. Red traces were induced by 60% current increment stimulation over the threshold level. *C*, STP as a function of two compound EPSCs before and after increasing the compound amplitude above the initial threshold value. Each connected pair of triangles represents one cell. *D*, The percentage of improvement of STP was linearly related to the degree of EPSC amplitude increment, independently of EPSC shape (r = .68, *p* = .001). Measurements were made with EPSCs of τ_rise_/τ_decay_ 0.3/3, 0.6/6 5/50, 5/83, 5/200 ms. *E*, The current peak amplitude at spike threshold for EPSCs with different kinetics. *F*, The total charge (open bars) and charge at spike threshold (filled bars) for EPSCs with different kinetics. High peak currents for fast EPSPs are partially due to the charging of the membrane capacitance. Charge was measured as the integral of the compound EPSC area (mean±s.e.m.; *n* = 10 cells).

To explore the influence of suprathreshold compound EPSC amplitude (which reflects the number of contributing synaptic inputs) on STP, we investigated how STP changed with increasing amplitude for EPSCs with a given kinetics (Figure 3*A, B*). The compound EPSC amplitude had a pronounced effect on STP. As previously described, slow compound EPSCs (τ_rise_/τ_decay_ 5/50 ms) induced spikes with low temporal precision at threshold amplitude. However, after increasing the compound EPSC amplitude by 60% above the threshold level, slow EPSCs elicited precise spike responses, with a STP of ≤2.57 ms (Figure 3*C*), comparable to what fast EPSCs produced at the threshold level. Changing the compound EPSC amplitude of fast EPSCs (τ_rise_/τ_decay_ 0.1/1 ms) had a much stronger effect on STP than amplitude changes for slow EPSCs: to increment STP by 50%, the fast compound EPSC amplitude had to be increased by only 5%, but the slow compound EPSC by as much as 40%. This suggests that, given the proper amplitude, both fast and slow compound EPSCs can produce temporally precise spike responses, but responses to fast EPSCs are much more sensitive to changes in EPSC amplitude, e.g. due to plastic changes in synaptic strength. As a general rule, increasing the compound EPSC amplitude increased spike temporal precision. As a consequence, slow EPSC compounds which only achieved poor STP at threshold amplitudes elicited precisely timed spikes when amplitudes were increased above threshold level.

To determine the relationship between the different compound EPSCs amplitudes and their kinetics in more detail, we examined the relationship between compound EPSC peak currents and charge at spike threshold amplitude for different compound EPSC kinetics. The average current amplitude of compound EPSCs necessary to trigger a spike at threshold level was maximal (mean = 3.3, s.e.m. = 0.04 nA; *n* = 10) for EPSCs with fast kinetics (τ_rise_/τ_decay_ 0.1/1 ms), and gradually decreased when the EPSCs became slower (Figure 3*E*). This suggests that the charge delivered by a compound EPSC might be an important parameter in the spike generation process. However, calculating the charge delivered for each compound EPSC at spike threshold showed that the slow compound EPSC (τ_rise_/τ_decay_ 5/50 ms) contained the highest charge (total: mean = 23.38, s.e.m. = 0.27 nC; at threshold: mean = 10.045, s.e.m. = 0.06 nC), and that charge was gradually reduced for faster compounds (Figure 3*F*). Hence, spike threshold could not be defined by charge or amplitude alone, but was clearly a function of EPSC shape, particularly of EPSC decay time. EPSC charge and amplitude at threshold level were a function of EPSC shape, with fast EPSCs triggering spikes at high current amplitudes but little charge, and slow EPSCs triggering spikes at low current amplitudes but high charge values. The high peak currents required for fast EPSCs to trigger spikes most likely reflect the current required to rapidly charge the membrane capacitance.

### The effect of temporal jitter in compound EPSCs on STP

**Figure 4:**
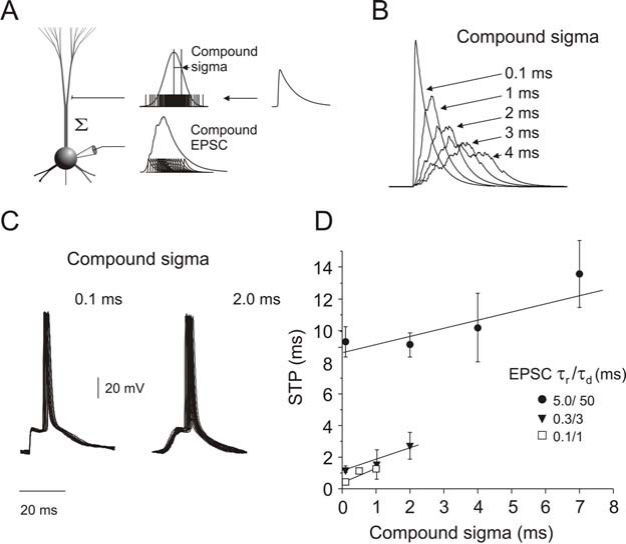
Spike time precision as a function of the temporal jitter of compound EPSCs. *A*, The diagram depicts the generation of a compound EPSC with a specific standard deviation (compound sigma) of the temporal distribution of EPSCs in the compound EPSC. *B*, Charge normalized compound EPSCs with different degrees of compound sigma but identical EPSC kinetics. *C*, Action potential responses induced by EPSCs τ_rise_/τ_decay_ 0.3/3 ms and different values of compound sigma. *D*, STP decreased with increasing temporal jitter in the compound EPSCs (*n* = 10), but the change was not significant. The two levels of spike precision exhibited by fast (*square* and *triangle*) and slow (*circles*) compounds matched the precision induced by the EPSC decay time shown in Figure 1*D*. Data were approximated with a linear regression (*solid lines*), slow compounds (*circles*) (r = .81, *p* = .18), fast compounds (*squares*) (r = .98, *p* = .09) *and* (*triangles*) (r = .98, *p* = .12).

To estimate the effect of input synchronization on STP, we varied the compound sigma in the EPSC volleys while keeping the number or EPSCs and thereby also the total charge per compound constant (Figure 4; cf. Methods). Due to the linear summation of EPSCs, larger values of compound sigma produced a “bumpy” compound shape (Figure 4*B*). STP decreased with increasing compound sigma, but the difference between spike responses induced by synchronized and unsynchronized compounds with identical EPSC kinetics was not significant (ANOVA: *p*>0.05, Figure 4*D*). Hence, even with temporal jitter in the compound EPSC, spike time precision was most strongly determined by EPSC shape.

### Differences in STP between responses induced by fast and slow EPSCs compounds are enhanced in the presence of background activity

**Figure 5:**
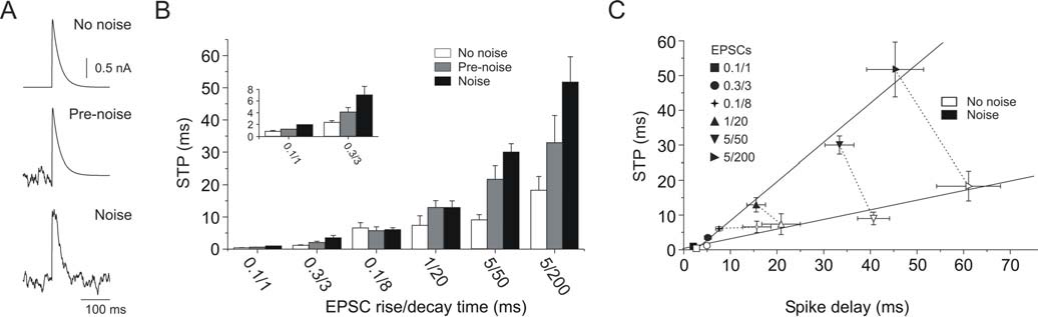
Differences in STP between fast and slow EPSCs increase with added synaptic background noise. *A*, Synchronized compound EPSC without noise (*top*), with the presentation of fluctuating currents previous to the compound (*middle*) and during the compound EPSC (*bottom*). *B*, Comparing the effect of noise on STP for different EPSC shapes. Inset shows results for EPSPs 0.1/1 and 0.3/3 drawn on a larger scale. The addition of background noise had similar effects on fast and slow EPSCs, with the exception of EPSPs with τ_rise_/τ_decay_ 0.1/8 ms, which produced similar STPs under all three conditions (ANOVA, *p*>0.05) and τ_rise_/τ_decay_ 1/20 ms, which showed decreased STP under noise conditions (*p*<0.05), but no difference in STP between the noise and pre-noise condition (*p*>0.05). For EPSCs with slow dynamics (τ_rise_/τ_decay_ 5/50 and 5/200 ms), STP was reduced in the pre-noise condition by 82.5±4.3% and by 182.7±8.5% in the noise condition. For fast EPSCs, STP was reduced by 39.5±1.7% in the pre-noise condition and by 127.9±3.2% in the noise paradigm (ANOVA = *p*<0.05). *C*, Spike delay relative to stimulus onset and spike time precision were linearly related. The addition of simulated synaptic background noise increased the slope of the STP vs. delay function (linear regression). Same data as in B, each symbol corresponds to the same EPSCs as B. Each connected pair of symbols corresponds to the same compound EPSC value (mean±s.e.m.; *n* = 10 cells).

We investigated the effect of background synaptic noise added to the membrane potential on STP. To address this issue, a fluctuating current mimicking stochastic synaptic input was linearly added to the compound EPSCs (Figure 5*A*; cf. Methods). The membrane potential fluctuations induced by the noise-current injection was mean = 15, s.e.m. = 0.53 mV (peak to peak) and did not elicit spikes when presented alone. The role of “synaptic” or “intrinsic” noise in spike timing was investigated by the injection of a fluctuating current in two different configurations: one during the 200 ms immediately preceding the synchronized compound EPSC onset (pre-noise case) and another, starting 200 ms before and continuing during the presentation of the EPSC (noise case) (Figure 5*A*). The rationale behind the pre-noise paradigm was to drive the population of voltage dependent ion channels into a more *in vivo* like distribution of activation states. In a slice preparation, the membrane potential fluctuates very little, presumably leaving all voltage dependent ion channels in a similar state. We found that fluctuating currents, presented before or during the compound EPSC generally reduced spike time precision (Figure 5*B*). For EPSCs with slow dynamics (τ_rise_/τ_decay_ 5/50 and 5/200 ms) the average STP was reduced by 82.5±4.3% in the pre-noise condition and by 182.7±8.5% in the noise condition. For fast EPSCs, STP was reduced by 39.5±1.7% in the pre-noise condition and by 127.9±3.2% in the noise condition. The statistical significance of the STP changes between the groups defined by the two noise conditions and the different EPSC shapes was evaluated using the analysis of variance between groups (ANOVA). A significant difference between no noise and pre-noise conditions was observed for EPSCs with τ_rise_/τ_decay_ = 0.1/1 and 5/50 (*p*<0.05) (Figure 5*B*). Differences between pre-noise and noise conditions were only significant for EPSCs with τ_rise_/τ_decay_ = 0.1/1 (*p*<0.05). A significant difference (*p*<0.05) was observed between the no noise and noise conditions for all EPSC shapes, except for EPSCs with τ_rise_/τ_decay _0.1/8 and 1/20.

There was a direct relationship between decreasing STP and increasing delay to spike occurrence (Figure 5*C*). High STP was associated with short spike delays (*n* = 10; *r* = 0.96, open symbols; *r* = 0.98, solid symbols). The addition of noise to the compound EPSCs increased the slope of the STP delay function both by shortening the spike delay and by decreasing STP (Figure 5*C*).

### Influence of sub-threshold currents on spike timing

To evaluate possible effects of spontaneous presynaptic inputs on STP we blocked excitatory and inhibitory synaptic transmission in a number of control experiments (*n* = 5 cells; cf. Methods). We found that STP was not affected by blocking synaptic inputs (data not shown), which consisted almost exclusively of miniature PSPs. Spontaneous spiking was rarely observed.

### Reliability of spike generation depends on the degree of synchrony in the EPSC compound and on background network activity

**Figure 6:**
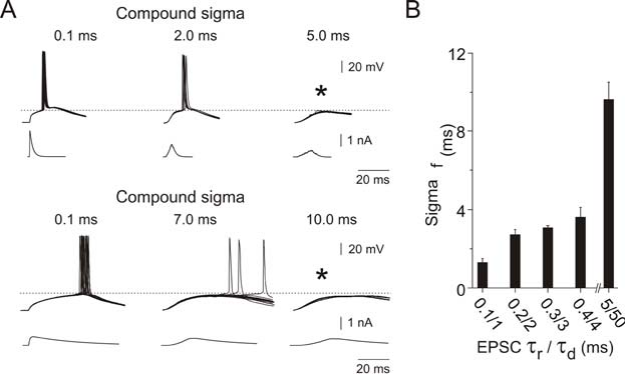
Reliability of spike generation as a function of input synchronization and EPSC shape. *A*, Spike responses to somatic injections of normalized compound EPSCs (τ_rise_/τ_decay_ 0.3/3 ms, *top traces*; τ_rise_/τ_decay_ 5/50 ms, *bottom traces*) with different levels of compound sigma. Compound sigma values for which spike responses failed to occur in 90% or more of the cases were termed “sigma_f_” (marked by the asterisks in *A*). *B*, Compounds with fast EPSC kinetics failed to elicit spikes when the temporal jitter in the compound exceeded a few milliseconds (sigma_f_≤4 ms, *n* = 4 cells). Spike responses to a compound EPSC with slow kinetics, on the other hand, were less sensitive to temporal jitter in the compound (sigma_f_ = 10±0.82 ms) (mean±s.e.m.).

In view of ongoing discussions about the importance of reliability in the propagation of synchronous spike activity for neocortical information processing (e.g. [3]) we investigated the reliability of spike firing as a function of input synchrony. We generated compound EPSCs with increasing degrees of temporal jitter, starting with synchronized compounds with a temporal jitter of 0.1 ms, i.e. 80% of the compound's EPSCs occurred within a time window of 0.1 ms (cf. Methods). To eliminate the effects of different spike thresholds in different neurons, all measurements started with the presentation of synchronized compounds with the current adjusted to the lowest amplitude at which spikes were elicited with 100% probability. This current was kept constant as the temporal jitter of the compound was successively increased, until spike responses were no longer elicited. As a quantitative measure for the reliability of spike generation (independent of temporal precision), we determined the compound sigma for which the spike response failure exceeded 90% (sigma_f_) as a function of the EPSC kinetics (Figure 6*B*).

As a general rule, compound EPSCs with small temporal jitter (i.e. small compound sigma) elicited spikes with a high probability. Increasing the compound sigma resulted in an increment of the number of spike response failures (Figure 6*A*). We found a significant difference between fast and slow compound EPSCs in their ability to reliably elicit spikes in the presences of input jitter. Slow compound EPSCs on average tolerated three times higher values of temporal jitter in their constituting input before spike responses began to fail. Spike generation in response to a slow compound EPSC (τ_rise_/τ_decay_ 5/50 ms) failed to trigger spikes at sigma_f_ = 9.6±0.89 ms (mean±s.e.m.). By contrast, fast compound EPSC inputs (τ_rise_/τ_decay_ 0.1/1, 0.2/2, 0.3,3 and 0.4/4 ms) failed to elicit spikes already at sigma_f_ = 3.6±0.46 ms (mean±s.e.m.; *n* = 10).

**Figure 7:**
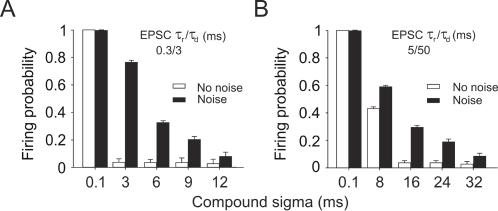
Reliability of spike responses to unsynchronized compounds is significantly increased by background synaptic noise. *A* Firing probability in response to a fast compound EPSC plotted versus different compound sigma values with and without background noise. *B* Firing probability in response to a slow compound EPSC plotted versus different compound sigma values with and without background noise. For both EPSCs tested, the addition of background noise resulted in a significant increase in firing probability for each compound sigma and - particularly for the fast compound EPSC - in a more gradual decline of firing probability with increasing compound sigma. Data shown are averaged across three cells.

Finally, we investigated the effect of synaptic noise on sigma_f_ for fast and slow compound EPSCs (Figure 7). We found that synaptic noise had no effect on sigma_f_ for highly synchronized compounds (sigma = 0.1 ms). However, for less well synchronized compounds, the addition of synaptic noise resulted in a significant increase in sigma_f_, i.e. making the neuron more likely to respond even to poorly synchronized input events. In the example presented in Figure 7, sigma_f_ increased from 3 ms to 12 ms for the fast compound EPSC (τ_rise_/τ_decay_ 0.3/3 ms; Figure 7*A*), and from 16 ms to 32 ms for the slow compound EPSC (τ_rise_/τ_decay_ 5/50 ms; Figure 7*B*). Also, with added noise, sigma_f _changed more gradually with increasing compound sigma compared to the abrupt changes measured with noise free compounds.

## Discussion

We have shown that the shape of postsynaptic potentials not only determines the temporal precision and reliability with which cortical pyramidal cells generate response spikes, but also how spike generation is affected by the amplitude and temporal jitter of population inputs and by the overall level of background synaptic activity. The most important shape defining parameter was the time constant of the decay phase. This has interesting functional implications since the decay time constant of EPSPs depends on several parameters: 1) the level of background activity, 2) the dendritic location of the synapses, and 3) the active and passive membrane properties of the postsynaptic neuron. Our findings suggest 1) that high levels of synaptic background activity reduce the postsynaptic impact of poorly synchronized population signals, but permit the propagation of precisely timed, i.e. highly synchronized signals, and 2) that precisely timed spike patterns may be preferentially communicated via proximal synaptic terminals and between neurons with small membrane time constants.

Individual neurons in the neocortex *in vivo* are constantly bombarded with synaptic inputs from the surrounding network. Understanding the translation of ongoing synaptic inputs to action potential output is essential for the understanding of information processing in the cortex. For simplicity it may be said that the neocortex exhibits two main types of spike response patterns: Besides the typically observed rate changes in response to sensory input or during behavioral tasks, there are now a number of studies reporting the occurrence of behavior related precisely timed spike activity which can occur independently of rate changes [2], [43]–[45]. A recent *in vitro* study has shown that precisely timed spike patterns also occur in slice preparations of the neocortex, suggesting that this form of activity may be a fundamental mode of operation of the neocortical network [46]. The time course of membrane potential fluctuations seems to play an important role in controlling the timing of action potential generation. *In vitro* studies have shown that precisely timed action potential firing in neocortical neurons can be triggered by rapid fluctuations of membrane potential [36], [39]. Furthermore, Azouz and Gray [4] have shown that the number of spikes generated in responses to a visual input *in vivo* scaled with the magnitude of the high frequency components in the sub-threshold membrane potential. Membrane potential fluctuations are a function of synaptic input currents and their time course. Here we investigated spike time precision as a function of EPSC shape, at threshold and suprathreshold amplitude, and of the degree of synchronization of population synaptic inputs. Furthermore, the influence of synaptic background noise and channel noise on spike timing was examined.

We used somatic current injection to induce membrane potential excursions similar to those induced by synaptic inputs reaching the soma. This approach does not allow any conclusions about dendritic processes of synaptic integration preceding the arrival of EPSCs at the soma. However, it does provide information about the somatic mechanisms of spike generation, which was the focus of the present study. An alternative approach to mimicking synaptic inputs would be to use dynamic current clamp, a technique that mimics conductance [47], [48] and takes into account that amplitudes of synaptic potentials are a function of the difference between the membrane potential and the reversal potential. This approach would have allowed to estimate the number of PSCs necessary to reach a certain voltage value. Yet another approach would be to actually elicit ‘true’ PSCs, e.g. by using photodynamic stimulation of selected presynaptic neurons [49]. However, since our measurements were all relative to spike threshold voltage, our conclusions are independent of the actual number of PSCs driving the membrane potential. Furthermore, direct control of EPSC shape was a priority in this study, resulting in the choice of the somatic current injection technique.

### EPSC shape and amplitude

Our main finding was that spike time precision, at threshold amplitude, strongly depended on the time course of EPSCs. Within the known physiological range of EPSC shapes tested here, the time constant of the rising phase varied between 0.1 and 23 ms, while the time constant of the decaying phase covered three orders of magnitude (1–250 ms). Our results show that primarily the decay time of an EPSP determines the temporal precision of spike responses (Figures 1,2). At threshold levels of depolarization, EPSCs with short decay phase triggered spikes with high temporal precision, whereas EPSCs with a long decay time triggered spikes with poor temporal precision. However, STP in response to slow EPSCs could be improved to almost match fast EPSC performance by increasing the amplitude of the population EPSC (Figures 3*C, D*). This amplitude increase, however, had to be quite significant (Figure 3*D*).

The link between decay time constant and spike timing precision seems to be via the time span the membrane potential spends close to or at the spike threshold level. This is the time window during which a spike can occur. This time window is short for fast and long for slow EPSCs. Consistent with this minimal explanation is the fact that the decay time constant and the EPSC half-width describe STP equally well (compare Figures 2*C and 2D*). The definition of a spike threshold, however, strongly depends on EPSC shape as well. In terms of peak voltage, fast EPSCs had a much higher peak than slow EPSCs (compare e.g. Figures 3*A, B, E*). By contrast, slow EPSCs required a much higher charge but a lower peak current to trigger a spike (Figures 3*E, F*). The only way of defining a spike threshold for the purpose of comparing EPSCs is via the probability of eliciting spikes. Our data suggest that at the threshold level, STP in layer V neocortical pyramidal neurons is quite sensitive to the time course of the membrane potential trajectory induced by different EPSC kinetics. However, differences in EPSC shapes matter less with increasing EPSC peak amplitude. At threshold level, however, fast and slow EPSCs have distinct spike triggering features: EPSCs with short decay time are characterized by high EPSC peak amplitudes and low charge, inducing spikes at short delays with high precision and reliability, and with little sensitivity of these parameters to noise. The opposite statement holds for threshold level EPSCs with long decay time.

### Effects of synaptic background noise

Synaptic transmission in the CNS is noisy, possibly as a result of the random activation of the postsynaptic terminals [4], [5], [20], [32]. This unpredictability of synaptic transmission is believed to induce a high degree of spike response variability [35], [40], [50]–[52]. Differences in spike response variability have been identified in simultaneous recordings from three successive stages of the visual system (retinal ganglion cells, thalamic relay cells and layer IV visual cortex, simple cells) [33]. Retinal cells responded in a more reliable way, while cortical cells responded much more variably. These studies suggest that the spike response variability may be the result of accumulated noise occurring at each of a number of stages of synaptic transmission. Our data suggest that intrinsic or channel noise also contributes significantly to spike response variability. The pre-noise presentation (Figure 5*A*), which has been shown to have a long-lasting effect on ion channel activation states [53], [54], resulted in a significantly reduced STP for EPSCs with τ_rise_/τ_decay_ = 0.1/1 and 5/50 (Figure 5*B*). Interestingly, this “intrinsic” or channel noise accounted for about half the average reduction in STP observed under the noise condition (compare grey and black bars in Figure 5*B*). This would imply that the membrane potential time course in the immediate past, i.e. the last 100–200 ms prior to PSC, affects STP just as much as the present event. The homogeneity of ion channel activation states is an often neglected but functionally relevant artifact of the slice preparation, resulting from the lack of network activity and the associated lack of membrane potential fluctuations.

On average, simulated synaptic background noise (“noise condition”) reduced the average STP by a factor of 2 to 3. Although the relative changes were similar for fast and slow EPSCs, in absolute terms the STP in response to fast EPSCs was altered more strongly by the presence of noise. For the fastest EPSCs tested (τ_rise_/τ_decay_ = 0.1/1 ms) there was a significant difference in STP between no noise and pre-noise condition (ANOVA, *p*<0.05). However, even in the presence of noise STP was still<2 ms (Figure 5*B*).

### Synchronization of inputs

Recent studies suggest that a temporal correlation of background synaptic inputs is required to produce the variability in spike firing typically observed *in vivo* [40], [55]. We investigated the effect of temporal jitter in the compound EPSC on the temporal precision of the spike response. Within the time windows tested here (compound sigma from 0.1 to 7 ms for slow and from 0.1 to 2 ms for fast EPSCs), the average spike time precision was reduced by increasing temporal jitter in the input but, surprisingly, the reduction in STP between sigmas was not significant (ANOVA test, *p*>0.05), with only one exception. STP was significantly reduced only in comparison between compound sigmas of 0.1 and 7 ms. Overall, although slow EPSCs performed less well in producing spikes with high temporal precision than fast EPSCs did, they triggered spike responses much more reliably than fast EPSPs when inputs were desynchronized (Figure 6). This is consistent with the findings of Grande et al. [56] who showed that spike responses in neocortical pyramidal cells to two simulated input spike trains were more sensitive to the degree of synchronization of inputs when presynaptic spikes were represented by short rather than by long EPSCs.

In conclusion, there seems to be a trade-off between temporal precision and reliability of spike generation. Signal transmission at spike threshold level with fast compound EPSCs results in temporally precise spike responses, but requires well synchronized input activity. By contrast, slow compound EPSCs reliably propagate signals that are less well synchronized but with more variability in spike timing. From this, to appears that fast EPSCs are likely to be the carriers of precisely timed spike patterns observed in neocortical activity [2], [43]–[45], whereas slow EPSCs are responsible for transmission of rate information which does not depend on temporal precision in the millisecond range. This assumption is supported by the findings of Grande et al. [56] who showed that the ability of a pyramidal cell to code input synchrony in its output spike rate is high if inputs are represented by fast EPSCs and low if inputs are represented by slow EPSCs.

It is important to mention in this context that the time course of EPSP decay can be shaped by active dendritic conductances [57] as well as by the level of background activity [20]–[22], [58]. High levels of background activity shorten the EPSP decay time by reducing the cell's input resistance and, hence, time constant. Thus, with high background activity the synaptic integration time window is shortened, leaving desynchronized inputs less effective, whereas highly synchronized inputs remain unaffected. Hence, even during periods of elevated background activity, highly synchronized input will effectively trigger postsynaptic spikes at high temporal precision. This may explain [3] how synchronous spike activity is passed on between large groups of neurons over many synaptic steps in the presence of high levels of background activity to produce the precisely timed spike patterns observed *in vivo* [2], [43]–[45] and *in vitro* [46].

Furthermore, inhibition may also play an important role in controlling spike temporal precision [59]. A sequential arrival of an excitatory input volley followed shortly afterwards by an inhibitory input wave would result in an abbreviated EPSP. The proper temporal correlation between excitation and inhibition may, thus, be a means to improve the temporal precision of spike generation in response to slow EPSCs.

Finally, the fact that the decay time constant of EPSPs also depends on the dendritic location of synapses and on the active and passive membrane properties of the postsynaptic neuron has another potentially important functional implication. It suggests that precisely timed spike patterns may be preferentially communicated via proximal synaptic terminals and between neurons with small membrane time constants.

## Material and Methods

### Experimental procedures

Sagittal neocortical slices (300 µm thick) were obtained from 23- to 28-day-old Sprague-Dawley male rats by using standard procedures. Animal treatment was according to the Freiburg University's and German guidelines on the use of animals in research. After being cut using a vibratome, the slices were incubated at 33°C for 1 hr, and then held at room temperature (20–22°C) until being transferred to the recording chamber. All experiments were performed at 35°C. Different bath solutions were used for the dissection, the incubation and the recording. The extracellular solution during the dissection contained 217 mM Sucrose, 2.5 mM KCl, 1.25 mM NaH_2_PO_4_, 7 mM MgCl_2_, 0.5 mM CaCl_2_, 25 mM NaHCO_3_ and 10 mM glucose, pH 7.4. For the incubation solution, the Sucrose was substituted by 125 mM NaCl, and Glucose was raised to 25 mM. For the recording solution we increased the CaCl_2_ to 2 mM from the incubation solution and reduced the MgCl_2_ to 1 mM. All solutions were saturated with 95% O_2_ and 5% CO_2_. Somatic whole-cell recordings from layer V pyramidal neurons were performed under visual control using infrared differential interference contrast (IR-DIC) optics. Cells were stained with 0.1% biocytin and their pyramidal shape and location in layer V were verified anatomically. Passive cell characteristics were determined by measuring the input resistance and membrane time constant.

Patch pipettes (5–8 MΩ) were filled with a solution containing 140 mM K-gluconate, 10 mM HEPES, 2 mM MgCl_2_, 2 mM NaATP, 10 mM EGTA, and 0.1% biocytin, (pH 7.3, adjusted with KOH). The liquid junction potential error was not corrected. Recordings were made in a current-clamp mode using an Axoclamp 2B amplifier (Axon Instruments, Foster City, CA). The voltage and current outputs were low pass filtered at 10 kHz and sampled at 20 kHz using a CED 1401 (Cambridge Electronic Design, UK). Control recordings were made in the presence of 10 µM CNQX (an AMPA/kainate-receptor antagonist). 100 µM APV (a NMDA-receptor antagonist) and 10 µM bicuculline methiodide (a GABA_A_-receptor antagonist) to block synaptic transmission and to exclude the influence of presynaptic inputs on our measurements.

### Compound EPSCs

Simulated EPSC time courses were computed using the beta-function:
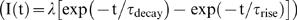
where λ is the single EPSC amplitude, *t* is time, and τ_rise_ and τ_decay _are the rise and decay time constants of the EPSC, respectively. The beta-function allowed the independent control of the rise and decay time constants of the simulated EPSCs. Compound EPSCs were generated by linear summation of 100 single EPSCs that were temporally distributed around a mean arrival time according to a Gaussian distribution. Standard deviation of the Gaussian, i.e. the temporal jitter of EPSCs arrival times in a compound, was systematically varied and termed “compound sigma”. Each compound EPSC consisted of mixtures of only one type or shape of EPSC.

The spike responses were evaluated by the repeated injection of the same compound EPSC for at least 500 times, with an inter-stimulus interval of 1 second. To make sure that the large number of repeated stimuli did not change the response properties of the cell, we compared STP values measured at the beginning and at the end of the set of 500 stimuli and monitored the cell's input resistance using hyperpolarizing currents injected after each 20^th^ stimulus. Also no prolonged spiking activity or burst firing were elicited by the repetitive compound EPSC injection. Cells which showed changes of input resistance, drifts in membrane potential or changes in spike response were excluded from the analysis.

### Synaptic background noise

In some experiments, “synaptic noise” was added to the current stimulus to mimic the voltage effects of ongoing background activity *in vivo*. Fluctuating currents were generated assuming inputs from independent groups of excitatory and inhibitory cells [40]. The total noise input to the cells was produced by adding two independent realizations of a Poisson process, convolved with either an EPSC or an IPSC (shot noise). The rates of the Poisson processes were 2 kHz for the excitatory and 1 kHz for the inhibitory input, reflecting the fact that excitatory neurons are at least twice as numerous as inhibitory neurons. Individual excitatory inputs were represented by a fast EPSC (0.3 ms τ_rise_ and 3 ms τ_decay_). The inhibitory PSCs delivered twice the charge of EPSCs with a 0.1 ms IPSC rise time and 6 ms decay time, keeping the mean injected current at 0 nA. Hence, noise injection did not contribute a DC component to the membrane potential. Compound EPSCs were linearly added to the background noise current that varied from trial to trial. Two different experimental paradigms were designed to investigate the role of background noise and cell memory in spike time precision. In the first paradigm, the injection of the synaptic noise started 200 ms before compound EPSC onset and continued for 700 ms, hence overlapping with the compound EPSC (noise paradigm). Summation of the compound EPSC and noise was linear. In the second paradigm, the noise was injected only during 200 ms prior to compound EPSC onset and stopped 0.1 ms before the EPSC onset (pre-noise paradigm). The number of stimulus repetitions and the inter-stimulus interval were as described before.

### Calibration

To compare spike responses across different neurons, all stimulus currents were adapted to each individual neuron by use of a standard calibration stimulus to define each neuron's spike threshold. This standard or calibration stimulus was a compound EPSC consisting of 100 single EPSCs with a Gaussian temporal jitter with a compound sigma of 0.1 ms. The calibration procedure (described in detail below) was of key importance for this study to allow comparison of close-to-threshold responses across a large number of pyramidal cells as different pyramidal cells had different spike thresholds. As a consequence, threshold EPSC amplitudes varied from neuron to neuron. Using fixed amplitude EPSCs would therefore not have provided meaningful results. Furthermore, because of the dependence of the spike threshold on the temporal derivative of the membrane potential, each EPSC shape had different threshold amplitude. High or low current amplitudes were required to trigger spikes with fast or slow EPSCs, respectively. The calibration stimulus was a compound EPSC consisting of 100 single EPSCs with a Gaussian temporal jitter with a compound sigma of 0.1 ms (which will be referred to as “synchronized”; EPSCs with a Gaussian temporal jitter larger than 0.1 ms which will be referred to as “unsynchronized”). The amplitude of this compound EPSC was manually increased up to the minimal value where spikes were elicited with a firing probability of 1 in at least 100 successive trials. This calibration protocol was applied for every cell and for every EPSC shape tested, as well as in the experiments using unsynchronized EPSCs and noise. When compound EPSCs with different jitter values were compared, calibration was performed using the corresponding synchronized compound EPSC. For the experiments with noise-current injection, the calibration was performed with the corresponding synchronized compound EPSC without noise. Importantly, different EPSC shapes or kinetics required different current amplitudes to reach the calibration spike threshold. As a consequence, EPSC shapes could not be compared independent of current amplitude. Figure 3*E* shows how, at the calibration threshold level, EPSC amplitude varied as a function of EPSC kinetics.
